# Chikungunya Virus, Cameroon, 2006

**DOI:** 10.3201/eid1305.061500

**Published:** 2007-05

**Authors:** Christophe N. Peyrefitte, Dominique Rousset, Boris A.M. Pastorino, Regis Pouillot, Maël Bessaud, Fabienne Tock, Helene Mansaray, Olivier L. Merle, Aurelie M. Pascual, Christophe Paupy, Aurelia Vessiere, Patrice Imbert, Patrice Tchendjou, Jean-Paul Durand, Hugues J. Tolou, Marc Grandadam

**Affiliations:** * Institut de Médecine Tropicale du Service de Santé des Armées, Marseille, France; † Centre Pasteur du Cameroun, Yaoundé, Cameroon; ‡ Centre Médico Social, Yaoundé, Cameroon; § Hôpital d'Instruction des Armées Begin, Clamart, France; ¶ Institut de Recherche pour le Développement, Yaoundé, Cameroon

**Keywords:** Chikungunya virus, alphavirus, Dengue viruses, arbovirus, Cameroon, dispatch

## Abstract

We report the isolation of chikungunya virus from a patient during an outbreak of a denguelike syndrome in Cameroon in 2006. The virus was phylogenetically grouped in the Democratic Republic of the Congo cluster, indicating a continuous circulation of a genetically similar chikungunya virus population during 6 years in Central Africa.

Chikungunya virus (CHIKV), formerly only an anecdotally described arbovirus, is now a worldwide public health problem (*1*). Recently, numerous cases of CHIKV infection have been reported from a major outbreak of febrile illness around the Indian Ocean, which included Comoros, Mauritius, Réunion Island (*2*,*3*), and southern India (*4*).

CHIKV is widely distributed in tropical Africa (*5*,*6*) and in Asia (*7*). In Africa, until 2000, the virus was described as endemic, perpetuated through a sylvatic cycle involving wild primates, humans, and mosquitoes of the genus *Aedes* (*8*,*2*). During the past 6 years, the urban cycle has also tended to play a role in Central Africa (*6*). Nevertheless, although recent serologic surveys suggest a high prevalence of *Togaviridae*, *Flaviviridae,* and *Bunyaviridae* (*9*,*10*), understanding of the circulation and effects of arboviruses in Cameroon remains imprecise. This lack of understanding may reflect confusion between arboviral infections and hyperendemic *Plasmodium falciparum* infection.

We report the first isolation, to our knowledge, of CHIKV in Cameroon. The virus was identified during an outbreak of a febrile syndrome in French soldiers in Douala and in patients from an urban medical center in Yaoundé. We also found evidence of cocirculation of CHIKV and dengue virus (DENV).

## The Study

In Douala, Cameroon, 2 sporadic cases of a denguelike syndrome were recorded in French soldiers (patients 1 and 2) on April 3 and May 22, 2006, respectively (Table). From the end of May through the end of July 2006, more cases of denguelike syndrome, which included fever, asthenia, maculopapular rashes, and arthralgia, were observed in Yaoundé. The number of patients who sought treatment at the Yaoundé Medical Center peaked in mid-June 2006. Blood samples were collected from 30 of the 40 patients who visited the medical center. The 30 patients’ ages ranged from 1 to 54 years. The delay between the onset of symptoms and the sampling ranged from 0 to 39 days with a median of 4 days (Table). All but 1 patient lived in Yaoundé, and none of these patients had a history of travel abroad or from Yaoundé. Nine patients were Cameroonian, and all other patients were from other countries; 15 patients were female. A blood sample from a 53-year-old woman who returned to France from Yaoundé was also received. All patients had negative results for *P. falciparum* according to rapid test (Core Malaria Pf, Core Diagnostics, Birmingham, UK) and thick smear examination.

**Table:** Characteristics of patients with febrile acute denguelike syndrome, Cameroon, 2006*

Patient no.	Sex/age	City	Symptom onset	Sampling date	Delay, days	IgM†	IgG†	PCR‡
1	M/35	Douala	3 Apr	7 Apr	4	Neg	Neg	Neg
1	M/35	Douala	3 Apr	11 May	21	Pos CHIK	Pos CHIKV	Neg
2§	M/36	Douala	22 May	23 May	1	Neg	Neg	Pos CHIK
3	F/54	Yaoundé	11 Jun	12 Jun	1	Neg	Neg	Neg
4	F/49	Yaoundé	10 Jun	13 Jun	3	Neg	Neg	Neg
5	F/42	Yaoundé	11 Jun	12 Jun	1	Neg	Neg	Neg
6	F/41	Yaoundé	7 Jun	7 Jun	0	Neg	Pos Flavi	Neg
7	M/38	Yaoundé	15 Jun	19 Jun	4	Neg	Neg	Neg
8	M/30	Yaoundé	18 Aug	19 Jun	1	Neg	Neg	Neg
9	M/21	Yaoundé	15 Jun	19 Jun	4	Neg	Neg	Neg
10	F/32	Yaoundé	21 Jun	22 Jun	1	Neg	Neg	Neg
11	F/22	Yaoundé	19 Jun	26 Jun	7	Pos CHIKV	Neg	Neg
12	F/53	Imported case	20 Jun	26 Jun	6	Pos CHIKV	Pos CHIK	Neg
13	M/42	Yaoundé	24 Jun	27 Jun	3	Neg	Pos Flavi	Neg
14	F/42	Yaoundé	18 Jun	28 Jun	10	Neg	Neg	Neg
15	F/27	Yaoundé	18 Jun	29 Jun	11	Neg	Pos Flavi	Neg
16	F/43	Yaoundé	26 Jun	30 Jun	4	Neg	Neg	Neg
17	M/31	Yaoundé	16 Jun	30 Jun	14	Neg	Neg	Neg
18	F/37	Yaoundé	22 May	30 Jun	39	Pos CHIK	Pos CHIK	Neg
19	F/45	Yaoundé	10 Jun	30 Jun	20	Neg	Neg	Neg
20	M/45	Yaoundé	22 Jun	30 Jun	8	Neg	Pos Flavi and CHIKV	Neg
21	M/1	Yaoundé	23 Jun	30 Jun	7	Neg	Neg	Neg
22	F/9	Yaoundé	10 Jun	30 Jun	20	Neg	Neg	Neg
23	M/4	Yaoundé	26 Jun	30 Jun	4	Neg	Neg	Neg
24	M/54	Yaoundé	4 Jul	6 Jul	2	Neg	Neg	Neg
25	F/45	Yaoundé	14 Jun	5 Jul	21	Neg	Neg	Neg
26	M/48	Yaoundé	24 Jun	6 Jul	12	Neg	Neg	Neg
27	M/20	Yaoundé	30 Jun	1 Jul	1	Neg	Neg	Neg
28	M/37	Yaoundé	4 Jul	11 Jul	7	Pos CHIK	Pos CHIKV	Neg
29	M/36	Yaoundé	9 Jul	11 Jul	2	Neg	Neg	Neg
30	M/33	Yaoundé	28 Jun	10 Jul	12	Neg	Neg	Neg
31	M/32	Yaoundé	10 Jul	12 Jul	2	Neg	Neg	Pos DENV
32	F/38	Yaoundé	16 Jul	17 Jul	1	Neg	Neg	Neg
33	M/45	Yaoundé	21 Jul	26 Jul	5	Neg	Neg	Neg

*IgM, immunoglobulin M; Neg, negative; Pos, positive; CHIKV, chikungunya virus; Flavi, flavivirus; DENV, dengue virus. †CHIKV isolation successful. ‡DENV, West Nile virus, Wesselsbron virus, Rift Valley fever virus, Bunyamwera virus, and CHIKV antibodies tested. §DENV, West Nile virus, CHIKV tested by real-time RT-PCR.

Serum specimens were tested for immunoglobulin M (IgM) and IgG antibodies specific for DENV, West Nile virus (WNV), Wesselsbron virus, Rift Valley fever virus, Bunyamwera virus, and CHIKV by IgM-antibody capture (MAC-ELISA) and IgG sandwich ELISA, respectively (*11*). A serum sample was consider positive if the optical density (OD) ratio of viral antigen to uninfected cells was >3. The presence of CHIKV, DENV, and WNV genomes was tested for by specific real-time reverse transcription PCR (RT-PCR) (*12*). Virus isolation on C6/36 and Vero cells was attempted on samples that were positive by RT-PCR (*11*).

The serologic follow up of patient 1 (Table) for a 3-week period detected seroconversion to a virus antigenically related to CHIKV virus (the OD ratios obtained with the second sample were >3) for IgM and IgG. A sample from patient 2 was obtained the day after the onset of symptoms, and no antibodies to all tested arboviruses were detected. However, the specimen was positive by real-time RT-PCR for CHIKV. The patient’s sample yielded CHIKV when cultured, and the envelope gene was partially sequenced (position 10,238–11,367, GenBank accession no. Bankit851776). The 1.2-kb sequence genetic analysis did not show any codon deletion or insertion when compared with other African CHIKV sequences available in the GenBank database (*3*,*6*,*8*). A high degree of identity was observed when the sequence was compared with the Democratic Republic of the Congo (DRC) strains isolated in 2000 (*6*). Paired identity ranged from 97% to 98.1% at the nucleotide level and from 98.7% to 99.3% at the amino acid level. The Cameroon isolate displayed a higher nucleotide divergence (paired identity ranging from 95% to 95.5%) when compared with the 2006 Réunion Island strains (*2*,*3*,*13*). However, amino acid sequences were highly conserved (99%–99.5%). The sequence identity among these isolates highlights their common origin and particularly the genetic stability of CHIKV despite the 6 years and the geographic distance from the DRC outbreaks. As shown in the phylogenetic tree (Figure), the CHIKV Cameroon strain clustered with DRC CHIKV strains with a high bootstrap value of 100. This genotype of CHIKV was closely related to strains from the Central African Republic and the 1982 Uganda isolate (*6*,*8*). The close genetic relationship suggests a continuous circulation of a homologous CHIKV population in Central Africa with a high degree of genetic stability. The genetic stability of the Central African CHIKV strains during 24 years, whether associated with epidemic or sporadic cases, highlights the peculiar importance of the few mutations detected in the recent Réunion Island isolates (*3*). This also suggests that the Central African strain CHIKV zone of circulation now includes India (*4*), the Indian Ocean, and Cameroon.

**Figure:**
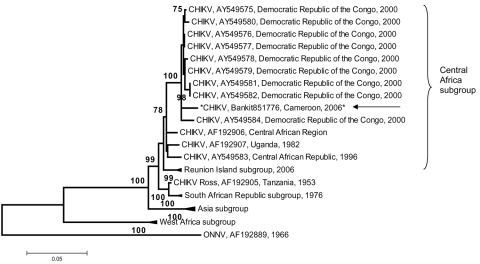
Phylogenetic tree of chikungunya virus (CHIKV) based on partial nucleotide sequences (3′ extremity of E1/3′-UTR, position 10,238–11,367). Phylogram was constructed with MEGA 2 program and tree drawing used the Jukes-Cantor algorithm for genetic distance determination and the neighbor-joining method. The percentage of successful bootstrap replicates (1,000 bootstrap replications, confidence probability >90%) is indicated at the nodes. The length of branches is proportional to the number of nucleotide changes (% of divergence). Asterisk (*) and arrow indicate the strains isolated in this work. The dark triangle corresponds to viruses clustering together. O’nyong-nyong virus (ONNV) sequence has been introduced for correct rooting of the tree. The GenBank reference no. for the Cameroon CHIKV isolate is EF051584.

The phylogenetic tree also illustrates the differences between the Cameroon isolates and the Asian subgroup isolates. Moreover, when compared with Asian CHIKV, including the 2006 isolates, the Cameroon strain showed 91%–91.9% and 96.8%–98.8% identity at the nucleotide and amino acid levels, respectively. Despite the similarity, cross-neutralization experiments must be conducted to confirm the protective effect of the Asian CHIKV-based vaccine against Central African strains (*2*).

Among patients from Yaoundé, 1 (patient 11) had only IgM antibodies specific to CHIKV, while patients 18 and 28 had both IgM and IgG antibodies specific to CHIKV (Table). One patient from Cameroon (patient 20) had IgG specific to both CHIKV and flavivirus. Three patients (nos. 6, 13, and 15), 2 of whom were Cameroonian, had antibodies specific for flavivirus. All samples were negative for WNV and CHIKV by RT-PCR. One sample (from patient 31) was positive for DENV; however, no virus was detected by cell culture. These results suggested a cocirculation of CHIKV and dengue virus during the same period, which is consistent with the suspected circulation of dengue virus, CHIKV, and yellow fever virus observed in a study from 2000 through 2003 in Cameroon (*9*).

In Cameroon, as in DRC (*6*), patients were likely infected in urban or periurban centers (Yaoundé, the capital of Cameroon; Douala, a major city). These infections occurred in a context where *Aedes albopictus* tends to replace indigenous *Ae. aegypti* in rural and urban Cameroonian environments (*14*). This finding suggests that urban cycles and urban vectors, in addition to the traditional forest-dwelling vectors, may play an important role in the maintenance and amplification of CHIKV in Africa.

## Conclusions

Since its first isolation in 1953 (*8*), CHIKV has been isolated in different Central African countries (*8*,*6*). Until now, only 2 alphavirus strains antigenically suspected to be CHIKV had been isolated from human patients in Cameroon (*15*). Recent serosurvey studies suggested a possible CHIKV circulation in Cameroon (*9*,*10*). Our Cameroon CHIKV isolate confirmed its circulation in this country. Our study suggests a 6-year continuous circulation of genetically stable and indigenous strains in Central Africa rather than importation of CHIKV from the recent Indian Ocean or Asian outbreaks. Moreover, the genetic stability of the Central African CHIKV highlights the importance of the unique molecular features that was shown in Réunion Island isolates (*3*).
